# Complete Genome Sequence of *Ureaplasma parvum* Serovar 3 Strain SV3F4, Isolated in Japan

**DOI:** 10.1128/genomeA.00256-14

**Published:** 2014-05-22

**Authors:** Heng Ning Wu, Yukiko Nakura, Daisuke Motooka, Shota Nakamura, Fumiko Nishiumi, Saki Ishino, Yasuhiro Kawai, Tatsuya Tanaka, Makoto Takeuchi, Masahiro Nakayama, Tomio Fujita, Itaru Yanagihara

**Affiliations:** a Department of Developmental Medicine, Research Institute, Osaka Medical Center for Maternal and Child Health, Osaka, Japan; d Pathology and Laboratory Medicine, Osaka Medical Center for Maternal and Child Health, Osaka, Japan; b Department of Infection Metagenomics, Genome Information Research Center, Research Institute for Microbial Diseases, Osaka University, Osaka, Japan; c Center for Medical Research and Education, Graduate School of Medicine, Osaka University, Osaka, Japan; e Fujita Clinic, Osaka, Japan

## Abstract

Here, we present the complete genome sequence of *Ureaplasma parvum* serovar 3, clinical strain SV3F4, isolated from a Japanese patient with a history of an infectious abortion.

## GENOME ANNOUNCEMENT

*Ureaplasma* species are bacteria belonging to the class *Mollicutes* (1). They are opportunistic pathogens that commonly inhabit the human urogenital tract (2); they lack a peptidoglycan layer (1) and hydrolyze urea (3). *Ureaplasma* species can be classified into two major groups: *U. parvum* (serovars 1, 3, 6, and 14) and *U. urealyticum* (serovars 2, 4, 5, and 7 to 13) (4, 5). *U. parvum* has been implicated in the morbidity and mortality of newborns (6–8). *U. parvum* serovar 3 is the most prevalent serovar detected in reproductive humans (9, 10). In this study, we present the complete genome sequence of *U. parvum* serovar 3, clinical strain SV3F4, isolated from a Japanese patient who had an infectious abortion during the 13th gestational week in her previous pregnancy.

A 10-kbp insert library of *U. parvum* was prepared and sequenced using the PacBio RS system (Pacific Biosciences, Menlo Park, CA). We obtained 45,965 filtered reads from one SMRT cell, with an average read length of 3,155 bp; these were assembled into one contig of 733,339 bp using the hierarchical genome assembly process (HGAP) (11). This contig was trimmed and circularized into the final complete genome of *U. parvum* strain SV3F4, including a 727,289-bp contig with a G+C content of 25.55%. A total of 571 predicted coding DNA sequences (CDS) and 6 rRNA and 30 tRNA genes were annotated by a BLAST-based search against the GenBank nt and nr databases.

A cursory search of the genome sequence revealed 7 sites at which urease was encoded and 28 sites at which transporters were encoded. These are consistent with the genome of *U. parvum* serovar 3 ATCC 700970. In addition, the genome encodes 5 putative virulence factors: urease (12), multiple-banded antigen (13), hemolysin (14), serine/threonine kinase, and protein phosphatase (15–17). The genome sequence reported in the present study is important for unraveling unknown cellular and/or pathophysiological mechanisms and may contribute toward developing future strategies for the treatment of ureaplasmal infections.

### Nucleotide sequence accession number.

The genome sequence for *U. parvum* strain SV3F4 has been deposited in the DDBJ database under the accession no. AP014584.
